# Mnemonic representations in human lateral geniculate nucleus

**DOI:** 10.3389/fnbeh.2023.1094226

**Published:** 2023-05-10

**Authors:** Masih Rahmati, Clayton E. Curtis, Kartik K. Sreenivasan

**Affiliations:** ^1^Department of Psychology, New York University, New York, NY, United States; ^2^Division of Science and Mathematics, New York University Abu Dhabi, Abu Dhabi, United Arab Emirates; ^3^Department of Psychiatry, Yale University, New Haven, CT, United States; ^4^Center for Neural Science, New York University, New York, NY, United States

**Keywords:** working memory, fMRI, modeling, human, retinotopy, saccades

## Abstract

There is a growing appreciation for the role of the thalamus in high-level cognition. Motivated by findings that internal cognitive state drives activity in feedback layers of primary visual cortex (V1) that target the lateral geniculate nucleus (LGN), we investigated the role of LGN in working memory (WM). Specifically, we leveraged model-based neuroimaging approaches to test the hypothesis that human LGN encodes information about spatial locations temporarily encoded in WM. First, we localized and derived a detailed topographic organization in LGN that accords well with previous findings in humans and non-human primates. Next, we used models constructed on the spatial preferences of LGN populations in order to reconstruct spatial locations stored in WM as subjects performed modified memory-guided saccade tasks. We found that population LGN activity faithfully encoded the spatial locations held in memory in all subjects. Importantly, our tasks and models allowed us to dissociate the locations of retinal stimulation and the motor metrics of memory-guided saccades from the maintained spatial locations, thus confirming that human LGN represents true WM information. These findings add LGN to the growing list of subcortical regions involved in WM, and suggest a key pathway by which memories may influence incoming processing at the earliest levels of the visual hierarchy.

## 1. Introduction

The lateral geniculate nucleus (LGN) of the thalamus is a key structure in the visual processing hierarchy, conveying information received directly from the retina to primary visual cortex (V1). The LGN shares several properties with early visual cortex, including a spatiotopic organization and sensitivity to contrast, motion, and color. Though the LGN has long been considered a simple feedforward relay station within the visual system (Jones, 1985; Sherman and Guillery, 2001), two pieces of evidence indicate that, in addition to external inputs, internal state may also play an important role in driving LGN activity. First, the majority of inputs to the LGN are feedback connections from V1 and other structures (Guillery, 1969; Sherman and Guillery, 1996), motivating the view that the LGN may be the earliest stage at which top-down input affects visual processing (Casagrande et al., 2005). Second, endogenous attention to both spatial location (O’Connor et al., 2002; McAlonan et al., 2008) and orientation (Ling et al., 2015) modulates LGN responses in a variety of ways that echo the effects of attention on activity in early visual cortex (Pessoa et al., 2003; but see Shah et al., 2022). Thus, the LGN appears to play an important role in attention by modulating early visual activity in accordance with task goals. In this study, we asked whether LGN similarly serves the temporary storage of mnemonic information in working memory (WM).

WM has traditionally been localized to higher-order cortical regions such as the prefrontal cortex (Wilson et al., 1993; Goldman-Rakic, 1995; Durstewitz et al., 2000; Lara and Wallis, 2015; Mendoza-Halliday and Martinez-Trujillo, 2017; Riley and Constantinidis, 2016; Leavitt et al., 2017) and posterior parietal cortex (Gnadt and Andersen, 1988; Constantinidis and Steinmetz, 1996; Todd and Marois, 2004; Bettencourt and Xu, 2016). However, there is growing support for the notion that WM involves representations arrayed in parallel across multiple cortical regions (D’Esposito and Postle, 2015; Curtis and Sprague, 2021). In addition to WM representations being distributed across cortex, recent findings suggest subcortical structures, including the cerebellum (Brissenden and Somers, 2019), superior colliculus (Shen et al., 2011; Dash et al., 2015; Sadeh et al., 2018; Rahmati et al., 2020), and basal ganglia (Postle and D’Esposito, 1999; Chatham and Badre, 2015), support WM maintenance and further underscore the distributed nature of WM (Christophel et al., 2017; Lorenc and Sreenivasan, 2021). Neuroimaging studies have also found WM related signals in the thalamus, but not in LGN (Rypma et al., 1999; Mayer et al., 2007). Despite little evidence directly implicating the LGN in WM, indirect evidence does exist and motivates the present study. Converging findings from human functional MRI (Harrison and Tong, 2009; Serences et al., 2009; Saber et al., 2015) and monkey electrophysiology (Supèr et al., 2001; van Kerkoerle et al., 2017) demonstrate that visual WM representations are encoded in the population activity of V1 neurons. Recent studies identified persistent activity both in the superficial and deep layers of monkey V1 (van Kerkoerle et al., 2017) and human V1 (Lawrence et al., 2018) during WM. Persistent activity in the superficial layers is not surprising given the long-standing belief that WM-related activity in V1 is the result of feedback signals from higher order cortical areas like PFC. The existence of persistent activity in the deep layers of V1, however, suggests that during WM delays this activity might be the source of feedback to downstream areas like the LGN.

**FIGURE 1:**
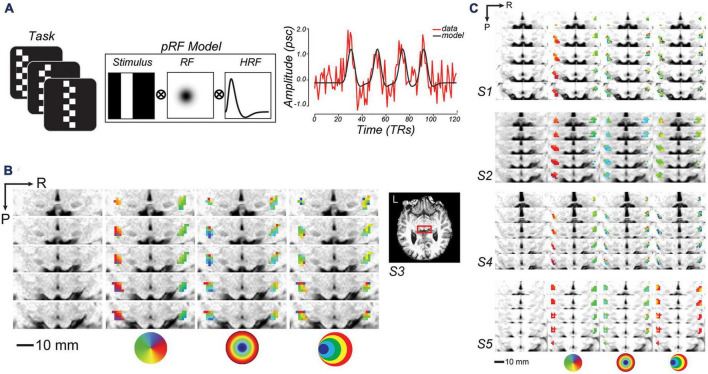
Topographic mapping of human lateral geniculate nucleus (LGN). **(A)** To model voxel population receptive fields (pRF), fMRI signal was collected while subjects viewed bars of contrast reversing checkerboards that swept across the visual field. Sweeping bar positions converted into binary apertures, were projected onto a 2D Gaussian model of a receptive field (RF), and convolved with a hemodynamic response function (HRF). To the right, a single sample voxel in LGN is plotted for one run. **(B)** Enlarged axial slices through the human LGN in an example subject (red box inset). R, right; P, posterior. From left-to-right, the columns depict the T1 anatomy, polar angle, eccentricity, and size parameter maps of an example subject (S3; thresholded at *r*^2^ ≥ 0.1). The colored circles are visual field keys. **(C)** Topography of LGN is consistent across other subjects.

Based on these findings, we predicted that LGN activity may store information about items encoded in WM through the sustained activation of neural populations tuned to task-relevant visual features. To test this hypothesis, we used inverted encoding models (IEMs; Brouwer and Heeger, 2009) and population receptive field mapping (pRF; Figure 1A; Dumoulin and Wandell, 2008) of human fMRI data collected while participants performed a demanding spatial WM task. These analytic approaches leverage well-described properties of the visual system to support inferences that are grounded in theoretically plausible mechanisms. We combined these approaches with a novel variant of a memory-guided saccade task that functionally dissociated visual stimulation from mnemonic information from motor preparation to overcome the inferential limitations of the sluggish hemodynamic response in order to pinpoint WM maintenance-related activity. To preview our results, we found that WM involved sustained activation of spatiotopically selective regions of LGN, providing novel evidence that the LGN is involved in WM maintenance.

## 2. Materials and methods

Portions of the data presented in this paper were previously reported as part of a study on the role of the SC in WM (Rahmati et al., 2020). Below we summarize the relevant methodological details.

### 2.1. Participants

Six volunteers (ages 27–49; one left-handed; one female) participated in this study. Subjects were healthy with no history of psychiatric or neurological disorders, had normal or corrected-to-normal visual acuity, and gave informed written consent. The study was approved by the New York University Committee on Activities Involving Human Subjects and the New York University Abu Dhabi Institutional Review Board.

### 2.2. Stimulus display

We controlled stimulus presentation using MATLAB software (The MathWorks, Natick, MA, USA) and Psychophysics Toolbox 3 (Brainard, 1997; Pelli, 1997). Stimuli were presented using a PROPixx DLP LED projector (VPixx, Saint-Bruno, QC, Canada) located outside the scanner room and projected onto a translucent display located at the end of the scanner bore that subtended ∼32^°^ of visual angle horizontally and vertically. Subjects viewed the screen at a viewing distance of 64 cm through a mirror attached to the head coil.

### 2.3. Eye tracking

**FIGURE 2:**
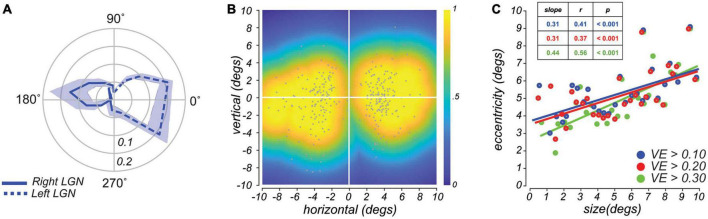
**(A)** Radial histograms of population receptive fields (pRF) polar angle in lateral geniculate nucleus (LGN) demonstrate strong contralateral coverage of the visual field. Based on pRFs from all subjects, the lines are the mean fractional volume representing each polar angle (±SEM). **(B)** Aggregate field of view (FOV) when pRF location and size parameters are combined. Each gray dot represents the center of single voxel pRFs. The color represents the maximum pRF value across the population of voxels in the LGN and reflects the relative effectiveness of visual stimulation in evoking a response in the LGN. **(C)** Size of voxel pRFs in the LGN increased linearly with eccentricity. Each dot represents a subset of voxels grouped according to their eccentricity (30 equally-distanced bins). Colors correspond to different variance-explained (VE) thresholds used for region-of-interest (ROI) selection. Lines, linear fit.

To ensure fixation compliance and to record saccadic responses, we measured eye position using an MRI-compatible Eyelink 2K (SR Research, ON, Canada). We preprocessed and scored eye-tracking data automatically, quantified the error (absolute Euclidian distance between the saccade landing point and the true target location), precision (average standard deviation of tangential and radial components of the saccade landing points) and response times of visual and memory guided saccades, and plotted example saccade trajectories shown in Figure 2B using the freely available iEye toolbox.^1^ Given the high degree of compliance with fixation during the delay (fixation breaks occurred in 0.5–2.0% of trials in four out of five subjects; the remaining subject made brief saccades away from and then back to fixation on 7.0% of trials), we did not exclude any trials from the fMRI analyses.

### 2.4. MRI data acquisition and preprocessing

Magnetic resonance imaging data were acquired in the Center for Brain Imaging at NYU with a 3-Tesla Siemens Prisma scanner (Siemens, Munich, Germany) using a 32-channel head coil. Twenty functional series of 120 volumes were collected for the retinotopic mapping task and twenty functional runs (except for one subject from whom we collected ten runs) of 232 volumes were collected for the spatial WM task. Each functional run was acquired with 14 coronal slices and a gradient echo, echo planar sequence with a 128 square matrix, 192 mm field of view, and 2.0 mm slice thickness, leading to a voxel size of 1.5 × 1.5 × 2.0 mm (TR = 1.5 s, TE = 41 ms, flip angle = 66^°^, bandwidth = 752 Hz/pixel). A partial Fourier factor of 7/8 was used to acquire an asymmetric fraction of k-space and GRAPPA parallel imaging with a factor of two was used to reduce acquisition time. The posterior edge of the acquisition volume was aligned in the mid-sagittal plane with the posterior edge of inferior colliculus. We also collected a high-resolution T1-weighted MPRAGE (0.8 mm isotropic voxels, 256 × 240 mm) in order to register functional scans to an anatomical image. In addition, for each scanning session we collected a single whole-brain-coverage functional image (TR = 10.8 s) with the same spatial resolution as the partial-brain coverage functional images to align the partial-coverage functional images to the whole-brain anatomical images. For preprocessing anatomical and functional fMRI data, we used FreeSurfer v6 and AFNI 17.3.0, respectively. We motion-corrected the functional data through a rigid body (six parameter) transform. After confirming that subject motion was limited (mean [max] rotation = 0.13^°^ [0.39°], mean [max] translation = 0.14 mm [0.50 mm]), we co-registered the functional data with the anatomical images according to the transformation calculated using the whole-brain-coverage functional image. All functional data was kept in the original spatial and temporal resolution (no smoothing) for both WM and retinotopy analyses. Finally, we removed the linear trend and converted the time-series to z-units for each voxel.

### 2.5. Population receptive field (pRF) mapping

We used established procedures to model the pRF parameters within voxels in LGN (Dumoulin and Wandell, 2008; DeSimone et al., 2015). Subjects viewed a black and white checkerboard-patterned bar whose elements reversed contrast with a full-cycle frequency of 8 Hz (Figure 1A). The bar subtended 8^°^ of visual angle across its width and extended beyond the boundaries of the screen along its length. The bar was oriented either vertically or horizontally and swept across the screen perpendicular to the bar orientation, passing through central fixation. Each scanning run consisted of four 30 s sweeps (left to right, right to left, top to bottom, and bottom to top) in a random order, with 12 s mean-luminance blank periods at the start and end of the run. Subjects performed a demanding fixation task that required them to map the color of the fixation cross (which could turn red, green, blue, or yellow every 1.5 s) to one of four button presses. We modeled each voxel in terms of a Gaussian pRF (Figure 1A; DeSimone et al., 2015, 2016). The pRF model provides a description of each voxel’s BOLD response in terms of a retinotopic location and extent. We also modeled the delay of the hemodynamic response function (HRF) and the baseline of the BOLD signal (DeSimone et al., 2015; Thomas et al., 2015). The delay parameter estimates the time to peak and time to undershoot of the HRF, while the baseline parameter ensures that the modeled and measured BOLD signals vary about a single global mean. In an initial phase of the parameter estimation, we used a sparse and coarse grid search with an effective stimulus downsampled by 2D bilinear interpolation to 5% of the original resolution. The best fit from the sparse sampling of model parameter space was used to estimate the best fitting HRF parameters (delay to peak and undershoot), and then used as a seed in the final phase of a fine-tuned gradient-descent error minimization using the non-resampled stimulus.

For each subject, we defined the LGN region-of-interest (ROI) as follows. First, we identified voxels with a spatiotopic organization using the pRF data with a model threshold of r^2^ ≥ 0.1 (Dumoulin and Wandell, 2008; DeSimone et al., 2015). Then, we applied an anatomical LGN mask based on a probabilistic atlas (Iglesias et al., 2018) using segmentation provided in FreeSurfer. The pRF model failed in 1 subject (S4), even when lowering the cutoff threshold, and we could not discern topography in LGN. Thus, for further analysis for this subject, we selected all voxels within the LGN based on anatomic T1 images as well as retinotopic data collected and pRF estimated for another independent study with a different protocol (Mackey et al., 2017). Importantly, our spatial WM results were not dependent on subject S4; indeed, the results were statistically more robust when excluding S4, although we include S4 in the results presented below for completeness. Using procedures similar to (Winawer et al., 2010; Mackey et al., 2017), we estimated the field of view (FOV) of the LGN map from the full pRF model. To represent the FOV of the full LGN map in visual space, we used 2D Gaussians whose positions within the visual field and widths were determined by each voxel’s pRF center and size parameters, and whose maximum value equaled 1. We did this on the pRF parameters aggregated across the left and right LGN of all subjects. Since many points in the visual field were covered by several pRFs, when combining the pRFs we mapped each visual field coordinate to the maximum pRF value.

### 2.6. Spatial working memory experiment

**FIGURE 3:**
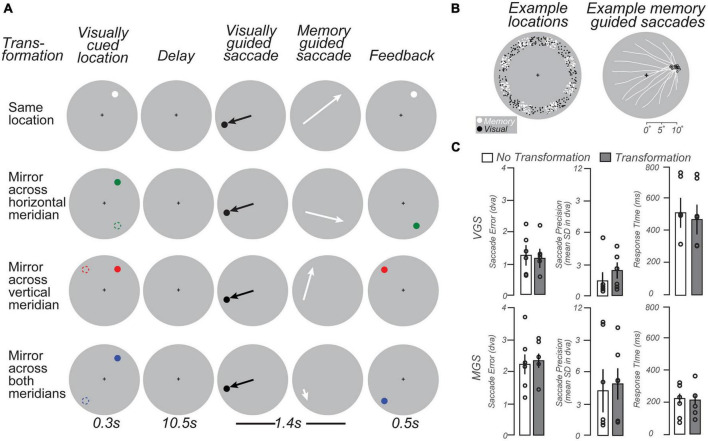
Working memory task schematic and behavioral data. **(A)** Schematic of four types of memory guided saccade (MGS) trials. In each condition, trials began with a brief visual target located in the periphery (colored dots; left column). Following a delay, subjects made a visually guided saccade (VGS) to a target whose location was unpredictable. Then, subjects immediately made a MGS to a location based on the initial visual target. In one condition, the MGS was directed to the visual target. In the other conditions, the MGS was made to simple geometric transformations of the visual target (dashed circles, left column; for reference here but not displayed). These included mirror transformations across each meridian and both meridians. The color of the visually-cued target indicated the type of transformation. Feedback was provided after the MGS with a visual stimulus at the correct location. Because of the VGS, the metrics of the MGS could not be predicted. The transformations dissociated the goal of the MGS from the visually-stimulated retinal position. **(B)** Left: Locations of VGS and MGS targets were distributed 9–11^°^ in the periphery. Right: MGS trajectories (white lines) from an example subject that converge on a single location. Note that the saccades start from a wide variety of peripheral locations following the VGS, but converge in these examples to MGS target locations (black circles) just above the horizontal meridian in the right visual field. **(C)** VGS were slightly more accurate and significantly slower than MGS. Bars = mean (± SEM).

We developed a modified delayed oculomotor response task (Figure 3A) to measure WM representations in the LGN. Each trial began with a brief visual stimulus (full contrast circle with a radius of 0.25^°^ of visual angle) presented for 300 ms in the periphery at one of eight angular locations evenly spaced from 22.5 to 337.5^°^ of polar angle in 45^°^ intervals and jittered by ±10^°^, at an eccentricity of 9–11^°^ of visual angle (Figure 3B, Left, white dots). The color of the visual stimulus indicated the transformation required to remap its location to the goal of a later memory-guided saccade (MGS). A white stimulus indicated no transformation; green indicated a MGS to the location mirrored across the horizontal meridian; red indicated a MGS to the location mirrored across the vertical meridian; and blue indicated a MGS to the location mirrored across both the horizontal and vertical meridians. After a 10.5 s memory delay, a black dot appeared for 400 ms at a random uniformly sampled polar angle (0–360^°^) and radius (9–11^°^ of visual angle) from central fixation (Figure 3B, Left, black dots). Subjects first made a visually-guided saccade (VGS) to this target, and then immediately made a MGS to the transformed location guided by memory (1.4 s allotted for both saccades). Finally, the stimulus was represented at the correct transformed location to provide performance feedback (500 ms). After subjects made a corrective saccade to the feedback target, an inter-trial interval (ITI) of 9.8 s of central fixation preceded the next trial. Each scanning run (10 per session) contained 16 trials (each 22.5 s), allowing us to sample each of the eight angular locations twice. The four MGS transformation conditions were counterbalanced across pairs of successive scanning runs, resulting in 80 trials per condition for four subjects (40 trials per condition for two subjects). Each scanning session lasted 58 min. All subjects practiced one block of 16 trials outside of the scanner and one or two blocks in the scanner before the experiment began.

Two task manipulations allowed us to uncover the nature of the information maintained in the LGN during the memory delay. First, the intermediate VGS prevented subjects from being able to plan, and potentially maintain, the metrics of the MGS during the delay. Second, the various transformations moved the task-relevant location — the goal of the MGS — to a position in visual space that was independent of the retinal position of the visual stimulus. Together, these manipulations served to eliminate simple visual and motor components while honing in on WM representations of visual space.

### 2.7. Average BOLD timeseries

**FIGURE 4:**
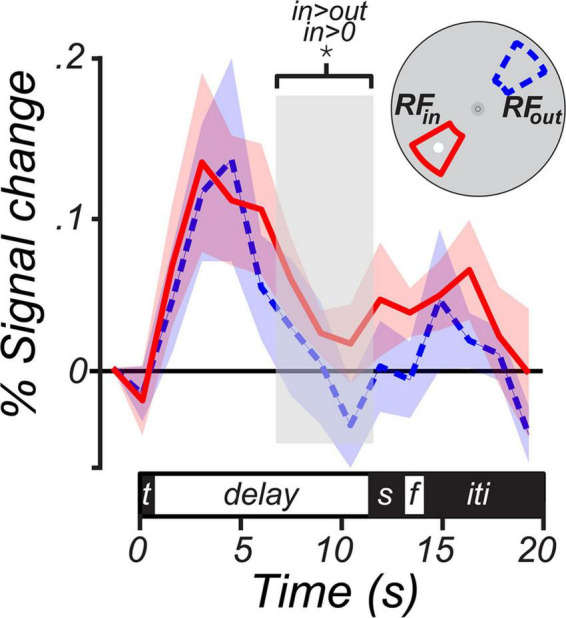
BOLD time courses from the human lateral geniculate nucleus (LGN) during working memory. The solid magenta line represents the group average (± SEM) BOLD signal from LGN voxels whose receptive fields were within a 30^°^ wedge centered at the MGS target (RF_in_). The dashed blue line represents the average (± SEM) from voxels whose receptive fields were within a 30^°^ wedge centered 180^°^ opposite the MGS target (RF_out_). The inset depicts the logic of the voxel definition based on the spatial selectivity of LGN voxels and the location of the MGS target. When the target was within the LGN receptive fields, BOLD signal persisted above the pretrial baseline during the memory delay (*p* < 0.01) and was significantly greater than the signal from the LGN voxels representing the visual field opposite of the target (*p* < 0.01). The delay period analyzed was limited to the last four TRs of the memory delay (light gray box). We choose this interval to isolate memory maintenance as it was long after the visual target and prior to the cue to generate saccades. The trial components on the time axis included the target (t), delay, visual and then memory guided saccades (s), feedback (f), and inter-trial interval (iti).

To measure the trial-averaged BOLD time course, we calculated mean BOLD response on each TR within a trial in two subsets of LGN voxels: RF_in_ voxels with a pRF-estimated polar angle within 30^°^ of MGS target and RF_out_ voxels with a pRF-estimated polar angle within 30°; of the opposite location (i.e.,180^°^) from the MGS target in that trial. We then averaged the resulting time course across trials separately for each voxel subset to produce the time course in Figure 4. Group-level significance of LGN activation during the memory delay was assessed by averaging the BOLD signal over the last 4.5 s of the delay period and performing a 1-sample (e.g., RF_in_ vs. baseline) or 2-sample (i.e., RF_in_ vs. RF_out_) *t*-test after a 1,000 iteration bootstrap across subjects.

### 2.8. Inverted encoding model (IEM)

To reconstruct a representation of spatial WM from the pattern of LGN activity during the memory delay, we used a spatial IEM (Brouwer and Heeger, 2009). First, we modeled each voxel’s response as a weighted sum of nine information channels, each in the form of a squared one-dimensional cosine function centered at one of the nine equally spaced polar angles around an invisible ring. We estimated voxel-channel weights by fitting a general linear model to a subset of data used only for training. For this training, we only used trials in which the visual stimulus and MGS target were co-located (“Same location” condition, Figure 1A). We then inverted these regression weights to estimate the contribution of each channel to a representation of visual-space in the held-out data from the other conditions that required a spatial transformation of the visual stimulus. Finally, we averaged all information channels, weighted by their estimated channel contribution, to reconstruct the population’s representation. We estimated the population activity in each trial by averaging each voxel’s BOLD activity during the last four TRs of the delay period. To increase the signal-to-noise ratio, we combined trials by computing a 2-fold mean trial timeseries, reducing the total number of trials by half while maintaining the counterbalancing of the exemplars across the memory locations. We repeated the IEM training and reconstruction procedure using a 2,000 iteration bootstrap procedure with different arrangements of trials for computing the 2-fold mean timeseries. This ensured that any effects were not simply due to bias in the sampling and recombination of trials.

### 2.9. Model assessment

We quantified the goodness of our reconstructions through the representational fidelity metric originally introduced by Sprague et al. (2016), which quantifies the similarity between a given reconstruction and a standard tuning function. We used a modified version of the fidelity which adjusts the over sensitivity of the fidelity to the gain of the reconstruction peak at the cost of sensitivity to deviations from the reconstruction center (Rahmati et al., 2020).

To validate the significance of our reconstructions, we built 2000 IEMs, each trained after shuffling the training data, and compared the fidelity distributions corresponding to the real and permuted data through a non-parametric Kolmogorov-Smirnov test at the individual subject level and a paired *t*-test, after a 1,000 iteration bootstrap across subjects, at the group level.

In order to link the pRF model of retinotopy and the spatial IEM, we compared each voxel’s polar angle preference derived from the two models. For the pRF model, we simply used the polar angle of the pRF center. For the IEM, we summed all information channels weighted by their estimated regression coefficients, yielding a polar angle tuning curve for each voxel. Since the IEM estimates were derived from the task in which all stimuli were 9–11^°^ in the periphery, we restricted our analysis to LGN voxels whose pRF centers were at least 5^°^ in eccentricity. We then calculated the circular correlation coefficient between the pRF polar angle and the peak of the IEM tuning curve.

## 3. Results

### 3.1. Retinotopic mapping

After measuring the LGN pRFs through our mapping procedures (Figure 1A), we overlaid the pRF model parameters on the T1 anatomical image to examine properties of the modeled pRFs (shown for a representative subject in Figure 1B and individual subjects in Figure 1C). We found orthogonal polar angle and eccentricity representations of the visual field along the LGN. The topography revealed a graded lower-to-upper visual field representation along the medial-to-lateral axis of the LGN, and a graded foveal-to-peripheral visual field representation along the ventral-to-dorsal axis. The LGN pRFs cover the visual field contralaterally (Figure 2A). We found a “bow-tie”-shaped distribution of polar angles, as found in previous fMRI studies of the retinotopy of the LGN (Schneider et al., 2004; DeSimone et al., 2015), which seems to imply an underrepresentation of angles near the vertical meridian. However, after we estimated the FOV of the LGN by considering the full receptive field model that combines the pRF centers and sizes, it is clear that most retinal locations have LGN representation (Figure 2B).

In addition, and similar to non-human primates (Casagrande and Norton, 1991; Xu et al., 2001), we found a positive correlation between the size and eccentricity of pRF parameters in the LGN (Pearson’s *r* = 0.50, *p <* 0.001; Figure 2C). This correlation value and the slope of this relationship increased as a function of voxel selection criteria (i.e., variance explained, VE). Overall, our model of the topographic structure of the human LGN closely resembles that of non-human primates (Malpeli and Baker, 1975; Xu et al., 2001) and previous reports in humans (Chen et al., 1999; Schneider et al., 2004; Kastner et al., 2006; Denison et al., 2014).

### 3.2. Spatial working memory

Subjects had similar accuracy, precision, and latency of visual and memory-guided saccades to previous studies that used delayed saccade or antisaccade tasks (Curtis and Connolly, 2008; Saber et al., 2015; Montez et al., 2017). This indicates that despite the transformations and double saccades, subjects could perform the task well (Figure 3C). Performance did not differ across task conditions (one-way RM ANOVAs, all *p*s > 0.4). The visually-guided saccades were slightly longer in latency than previous reports (c.f., Curtis and Connolly, 2008), perhaps owing to the long delay, complexity of the task, or the simultaneous task of remembering the future memory-guided saccade target.

The average BOLD signal in LGN voxels with RFs that overlapped the MGS target persisted above pre-trial baseline during the memory period [percent signal change; mean = 0.051, 95% CI = (0.016 0.087), one-tailed Student’s *t*-test, *p <* 0.01; RF_in_ in Figure 4]. In contrast, there was no evidence for persistent activity during the memory delays in voxels tuned to locations opposite the MGS target (RF_out_ in Figure 4). Memory-related activity was significantly greater for RF_in_ relative to RF_out_ voxels [percent signal change; mean = 0.04, 95% CI = (0.001 0.079), one-tailed Student’s *t*-test, *p <* 0.05]. This finding indicates (i) that neural activity in the LGN persists during the temporary retention of spatial information despite the lack of visual stimulation, and (ii) that memory-related persistent activity in the LGN is spatially specific.

**FIGURE 5:**
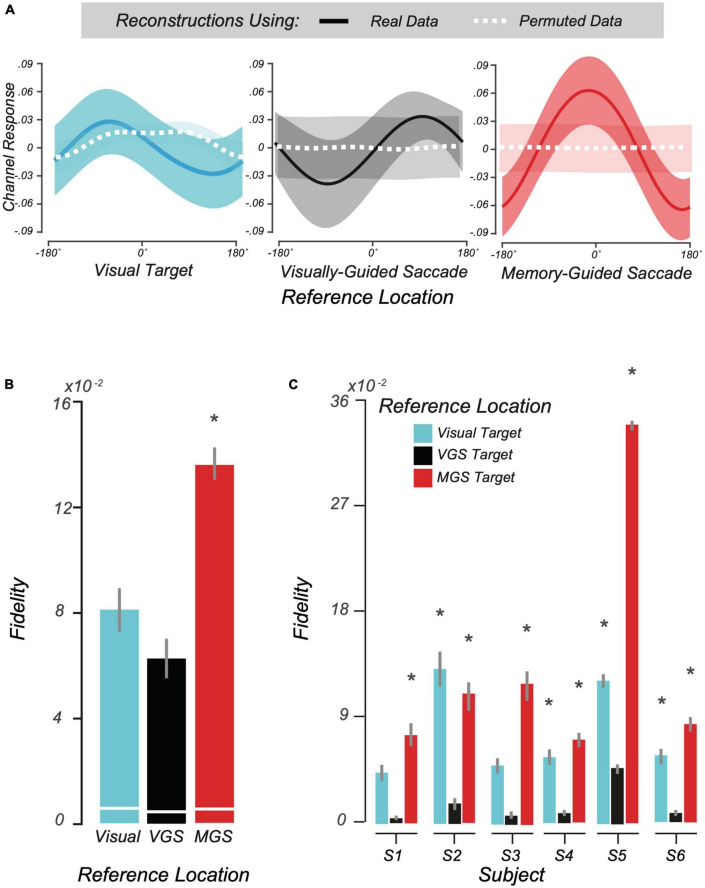
Modeling working memory (WM) representations in human lateral geniculate nucleus (LGN). **(A)** We used the second half of delay period activity in human LGN to reconstruct visual space. From left to right: the average reconstructed WM representation (± SEM) in visual space aligned to the visually-cued target, visually guided saccade (VGS) target, and memory guided saccade (MGS) target locations, respectively. In each panel, all trials are aligned to the corresponding reference location centered at 0^°^. The dashed white lines depict reconstructions from BOLD data with the trial labels permuted. **(B)** Representational fidelity (± SEM) corresponding to three reference locations, compared to shuffled data (white lines) computed at the group level. Note that the LGN population activity during the delay is largely tuned for the visual-spatial location of the MGS (*p <* 10^– 15^), not the visual target or VGS. **(C)** Even at the individual subject level, we find greater fidelity for the MGS location for all subjects. In one subject there was smaller tuning for the visually-cued target, but this small effect was not significant at the group level.

Motivated by the above trial-averaged BOLD data and our pRF findings, we used a multivoxel model of visual space, the IEM (Sprague and Serences, 2013; Rahmati et al., 2018, 2020), to test whether topographic patterns of activity in human LGN encode locations held in WM. Conceptually, IEM enables us to map a multivoxel population response into the coordinates of visual space. We assumed an underlying neural architecture based on the retinotopic organization of the voxels within LGN and modeled each voxel’s response with a set of basis functions that tiled polar angle space. Next, we tested the IEM model trained on no-transformation trials on trials that required transformations (see section “2 Materials and methods”). Consistent with the notion that LGN population delay activity encodes spatial information in WM, our model was able to accurately reconstruct the transformed location of the MGS (Figure 5A, Right). Importantly, these locations stored in WM were computed from spatial transformations of the visual targets and thus were not locations that were retinally stimulated during stimulus presentation. Models trained on the location of the visual target or the VGS location were unable to reconstruct these locations (Figure 5A, Left and center), indicating that LGN delay activity encoded the abstract representation of the memory location rather than the visually presented targets. Quantification of these results using our modified representational fidelity metric confirmed that LGN population activity during the delay was spatially tuned only for the location of the MGS target [subject mean fidelity = 0.13, 95% CI = (0.03.018), one-tailed Student’s *t*-test between real and permuted reconstructions, *p* < 0.01; Figure 5B]. Emphasizing the strength of this finding, we observed significant fidelity at the MGS location at the individual level in every subject (Figure 5C). Overall, the results were consistent and provide robust evidence for spatial WM encoding in topographically-organized human LGN.

## 4. Discussion

The LGN occupies the earliest stage in the brain’s visual hierarchy, and thus has the potential to influence incoming processing of the external visual world based on our internal state (Koch and Ullman, 1985). Motivated by evidence that visual WM activity selectively activates layers of V1 that receive top-down projections from higher visual regions as well as layers that project to LGN (van Kerkoerle et al., 2017; Lawrence et al., 2018), we hypothesized that LGN population activity encodes information about locations stored in spatial WM. We employed model-based neuroimaging analyses based on the known topography of visual field maps to reconstruct spatial WM representations in retinotopically organized LGN. Moreover, we found that LGN activity contained spatial WM information independent of retinal stimulation or planned saccades. This work adds to the growing list of subcortical regions - particularly the thalamus (Watanabe and Funahashi, 2012)–that represent WM-relevant information (Sreenivasan and D’Esposito, 2019). Previous studies of WM and the thalamus have largely focused on the mediodorsal nucleus (Funahashi, 2013; Peräkylä et al., 2017), which has reciprocal connections with the prefrontal cortex. The present study makes a novel contribution to this literature by demonstrating that a retinotopically organized portion of the thalamus–the LGN–encodes representations of space in support of WM. Intriguingly, this finding is consistent with evidence that representations stored in visual WM can bias visual input at early stages of processing (Soto et al., 2008; Kiyonaga and Egner, 2013).

### 4.1. Retinotopy in human LGN

The retinotopic representations we observed in LGN accord well with previous fMRI work. Specifically, we found a contralateral organization with a transition from lower to upper visual field representation reflected in superior to inferior regions of LGN (Chen et al., 1999; Schneider et al., 2004). We also found that foveal-to-peripheral representation was organized from posterior to anterior, although the foveal representation was inconsistent in its location along the inferior-superior axis across subjects. When considering the estimated RF centers, we replicated the previous observation that the vertical meridians are underrepresented (i.e., a bowtie-shaped distribution of RF centers across the visual field; shown in the distribution of dots in Figure 2B; Schneider et al., 2004). However, when we accounted for the full extent of the estimated pRFs (color map in Figure 2B), we found that voxels in LGN represented the entire visual field.

### 4.2. Working memory representations in LGN

In order to identify WM representations in LGN, we used a spatial IEM, which is a well-established method to derive neural representations from BOLD fMRI activity. Previous studies have employed similar models to describe WM storage in PFC and PPC (Ester et al., 2015), visual cortex (Ester et al., 2013; Rahmati et al., 2018), and SC (Rahmati et al., 2020). We show that models trained on delay period activity were able to reconstruct the memory target location (but not the visual stimulation or the visual saccade target), suggesting that the population activity in the human LGN encodes spatial representations of task-relevant mnemonic information that are abstracted from retinal stimulation or motor intention. Importantly, our ability to successfully model and decode spatial WM representations likely depended on the retinotopic organization of the LGN, which we verified with independent models of the receptive fields of voxels in LGN. Leveraging the topographic organization of the LGN and the receptive fields of individual voxels, we demonstrated spatially-selective persistent activity during WM maintenance (Figure 4). Together, these findings indicate that WM information in LGN is sustained via selective activation of spatially organized neural populations. The present results echo our previous report from this dataset that topographically-organized SC voxels represent WM information (Rahmati et al., 2020). An important question is whether and to what degree the nature of WM representations in LGN, a structure typically associated with visual perception, differ from those in SC, a region largely involved in visuomotor activity. Future studies that manipulate the visual features of the memoranda (e.g., spatial frequency) and the intended behavioral output (e.g., the type of motor response required) in concert with intervening visual stimulation or attention demands may be able to disentangle the roles of these and other subcortical regions in WM.

One plausible mechanism by which the LGN may represent spatial WM information is through population-based codes, such as attractors, that have been proposed to underlie mnemonic coding of visual space in the PFC (Compte et al., 2000; Khona and Fiete, 2022). Coherent mnemonic representations of space across thalamus and visual cortex may be organized by LFP oscillations at the alpha (8–13 Hz) frequency, which are thought to originate in LGN and facilitate visual thalamocortical communication (Lorincz et al., 2009; Hughes, 2011; but see Halgren et al., 2019). In line with this notion, the pattern of alpha oscillations over posterior cortical EEG electrodes has been used to reconstruct spatial WM representations (Foster et al., 2016), suggesting a potential link between alpha, the LGN, visual cortex, and spatial WM.

An important question is precisely how spatiotopic WM representations emerge in LGN. There are several lines of evidence that suggest that top-down influences from visual areas modulate LGN activity. For example, attention enhances feedback signals from V1 to LGN (Mock et al., 2018) while optogenetic activation of corticogeniculate neurons decreases response gain variability and increases information coding in LGN (Murphy et al., 2021). Most relevant to the current study, van Kerkoerle et al. (2017) recorded multi-unit and LFP activity in primate V1 during a WM task and found that sustained information about WM items was encoded in superficial and deep layers, indicating that WM signals in V1 represent feedback from cortex and feedback to LGN, as opposed to feedforward signals from LGN. The cascade of influences from higher visual regions (and beyond) to V1 and on to LGN may serve to improve the efficiency with which we can prioritize processing of spatial locations that are relevant to our internal goals (Koch and Ullman, 1985). At the same time, we cannot rule out the possibility that mnemonic representations in LGN are at least partially shaped by extracortical influences; for example, previous work has identified the thalamic reticular nucleus (TRN) as an early source of attentional modulations in LGN, consistent with the finding that attentional effects are larger in LGN than in V1 (O’Connor et al., 2002). While simultaneously imaging the LGN and visual cortex at high resolution presents a technical challenge that was beyond the scope of the present study, future studies that are designed to directly compare WM activity in visual cortex and LGN can help uncover the degree to which LGN WM representations are directly shaped by visual cortical activity.

## Data availability statement

The datasets presented in this study can be found in online repositories. The names of the repository/repositories and accession number(s) can be found below: Experimental data and analysis code are available at https://osf.io/ezn9j/.

## Ethics statement

The studies involving human participants were reviewed and approved by New York University Committee on Activities Involving Human Subjects and the New York University Abu Dhabi Institutional Review Board. The participants provided their written informed consent to participate in this study.

## Author contributions

KS, CC, and MR devised the experiment and wrote the manuscript. MR analyzed the data. All authors contributed to the article and approved the submitted version.

## Footnotes

1
github.com/clayspacelab/iEye
